# [Retracted] microRNA‑146b promotes neuroblastoma cell growth through targeting NUMB

**DOI:** 10.3892/etm.2024.12424

**Published:** 2024-02-13

**Authors:** Xiao-Li Ma, Xiao-Juan Zhang, Qian Du, Xiang-Nan Zhang, Shu-Yan Zhang, Huan-Fen Zhao

Exp Ther Med 19:3531–3536, 2020; DOI: 10.3892/etm.2020.8623

Following the publication of this paper, it was drawn to the Editor’s attention by a concerned reader that certain of the Transwell invasion assay data shown in Fig. 4C on p. 3535 were strikingly similar to data appearing in different form in other articles written by different authors at different research institutes that had either already been published, or were submitted for publication at around the same time; moreover, there were a pair of duplicated data panels within Fig. 4C itself, such that data that had been derived from the same original source had been used to portray the results of what were intended to have been differently performed experiments.

Owing to the fact that the contentious data in the above article had already been published prior to its submission to *Experimental and Therapeutic Medicine,* the Editor has decided that this paper should be retracted from the Journal. The authors were asked for an explanation to account for these concerns, but the Editorial Office did not receive a reply. The Editor apologizes to the readership for any inconvenience caused.

